# Study on Dry Sliding Wear and Friction Behaviour of Al7068/Si_3_N_4_/BN Hybrid Composites

**DOI:** 10.3390/ma14216560

**Published:** 2021-11-01

**Authors:** Kumar Subramanian, Sakthivel Murugesan, Dhanesh G. Mohan, Jacek Tomków

**Affiliations:** 1 Department of Production Engineering, Government College of Technology, Coimbatore 641013, India; kumar.sgm@gmail.com; 2 Department of Mechanical Engineering, Anna University Regional Campus Coimbatore, Coimbatore 641047, India; sakthi_vel_m@yahoo.com; 3 Institute of Materials Joining, Shandong University, Jinan 250061, China; 4 Faculty of Mechanical Engineering and Ship Technology, Gdańsk University of Technology, 80-233 Gdańsk, Poland; jacek.tomkow@pg.edu.pl

**Keywords:** dry sliding wear, silicon nitride, boron nitride, ANOVA, hybrid composites

## Abstract

Hybrid aluminium metal matrix composites have the potential to replace single reinforced aluminium metal matrix composites due to improved properties. Moreover, tribological performance is critical for these composites, as they have extensive application areas, such as the automotive, aerospace, marine and defence industries. The present work aims to establish the tribological characteristics of Al7068/Si_3_N_4_/BN hybrid metal matrix composites prepared by stir casting route and studied using a pin-on-disc apparatus under dry sliding conditions. The hybrid composite samples were prepared at various weight percentages (0, 5, 10) of Si_3_N_4_ and BN particles. To investigate the tribological performance of the prepared composites, the wear experiments were conducted by varying the load (20, 40 and 60 N), sliding velocity (1.5, 2.5 and 3.5 m/s) and sliding distance (500, 1000 and 1500 m). Wear experimental runs were carried out based on the plan of experiments proposed by Taguchi. The minimum wear rate was found with the composite material reinforced with 10 wt. % of Si_3_N_4_ and 5 wt. % of BN. Analysis of Variance (ANOVA) was employed to analyse the effect of process parameters on wear rate and coefficient of friction (COF). The ANOVA test revealed that the weight fraction of Si_3_N_4_ has more of a contribution percentage (36.60%) on wear rate, and load has more of a contribution percentage (29.73%) on COF. The worn-out surface of the wear test specimens was studied using its corresponding SEM micrograph and correlated with the dry sliding wear experiment results.

## 1. Introduction

Aluminium alloys have gained popularity in many industries owing to their light weight, excellent specific strength and high corrosion resistance [1,2]. However, their usage is limited in certain applications due to their poor wear resistance [3]. To improve their tribological as well as mechanical characteristics, various hard materials like silicon carbide (SiC), alumina (Al_2_O_3_), boron carbide (B_4_C), etc., have been tried as reinforcing agents in recent decades to develop aluminium-based metal matrix composites [4,5,6]. However, the addition of hard reinforcement decreases the machinability of the base alloy. To overcome this issue, soft materials such as graphite, MoS_2_ and BN are used as secondary reinforcement to develop aluminium metal matrix composites (AMMC) with multiple reinforcements (hybrid composites) [7,8,9]. The addition of multiple reinforcements will improve the various properties of the base aluminium alloy [7].

Al7068 exhibits higher mechanical strength compared to various other aluminium alloys, which makes it seamless for this research work, as most of its applications are related to the automobile field, such as connecting rods, motorcycle gears, etc. [10,11]. Further, improving its mechanical and tribological properties may replace some heavier metals in many industrial applications.

Silicon nitride (Si_3_N_4_) is preferred as the primary reinforcing material for the development of AMMCs owing to its excellent combination of mechanical, thermal and chemical properties, such as excellent wear resistance, corrosion and oxidation resistance, high hardness and good thermal shock resistance [12]. Moreover, some researchers have found that the wear resistance of Si_3_N_4_ reinforced AMMC is much higher than that of the corresponding unreinforced alloy [13,14,15,16]. Hexagonal boron nitride (h-BN), soft, and most stable among BN polymorphs, has excellent lubricating properties. It also possesses properties such as low density, excellent corrosion and wear resistance [17].

The stir casting process is frequently used for both ferrous and nonferrous components, and it is a reliable process [18]. Despite the different manufacturing methods of AMMCs, the stir casting technique was adopted by many researchers because of its commercial success in the production of large-sized components [19,20]. Moreover, the stir casting process has been proved as a simple and effective process for particle bonding due to stirring action during the addition of ceramic particles.

Birari and Shelke [9] investigated the tribological behaviour of hybrid composites of Al7068 alloy reinforced with boron carbide and graphite. The investigation found that the applied load and the weight fraction of reinforcing elements were the major influencing parameters for the wear and friction behaviour of the developed composites. Arul Daniel et al. [7] studied the influence of various process parameters on wear rate and friction coefficient of AA5059/SiC/MoS_2_ composites by employing Taguchi’s experimental design and ANOVA. The authors observed that the wear rate increased with the increase in sliding speed and sliding distance. Khatavkar et al. [21] studied the effect of h-BN on Al2024/hBN composites’ wear behaviour. The authors found that the wear resistance of the composite material improved up to 6% h-BN composition. Ul Haq and Anand [22] fabricated Al7075/Si_3_N_4_ composites using the stir casting process and studied the wear behaviour of the fabricated composites. The study results concluded that the micro-hardness and wear resistance of the composites improve with the addition of Si_3_N_4_ particles. Ul Haq and Anand [23], in another study on Al7075/Si_3_N_4_/Gr, observed that the wear resistance of the composite material initially increased with the addition of graphite particles up to 4 wt%; after that, it decreased. In their experimental study, Radhika et al. [24] concluded that Al/9%Al_2_O_3_/3%Gr composite has more wear resistance than the corresponding unreinforced alloy.

The main aim of this research work is to develop Al7068/Si_3_N_4_/BN hybrid AMMCs with enhanced tribological characteristics. The Taguchi plan of experiments and ANOVA were employed in this work to study the influence of five parameters, namely, weight % of Si_3_N_4_, weight % of BN, applied normal load, sliding velocity and sliding distance on the tribological behaviour of the fabricated Al7068/Si_3_N_4_/BN composites.

## 2. Materials and Methods

### 2.1. Materials

Al7068 was chosen as a base matrix material for developing composite materials. The chemical composition of Al7068 was identified through Energy Dispersive Spectroscopy (EDS). Table 1 shows the chemical composition of Al7068. Silicon Nitride (Si_3_N_4_) and hexagonal Boron Nitride (BN) particles were used as the reinforcements for the composite development. The sizes of the Si_3_N_4_ and BN particles were 10 μm and 5 μm, respectively.

### 2.2. Composite Fabrication

**Figure 1:**
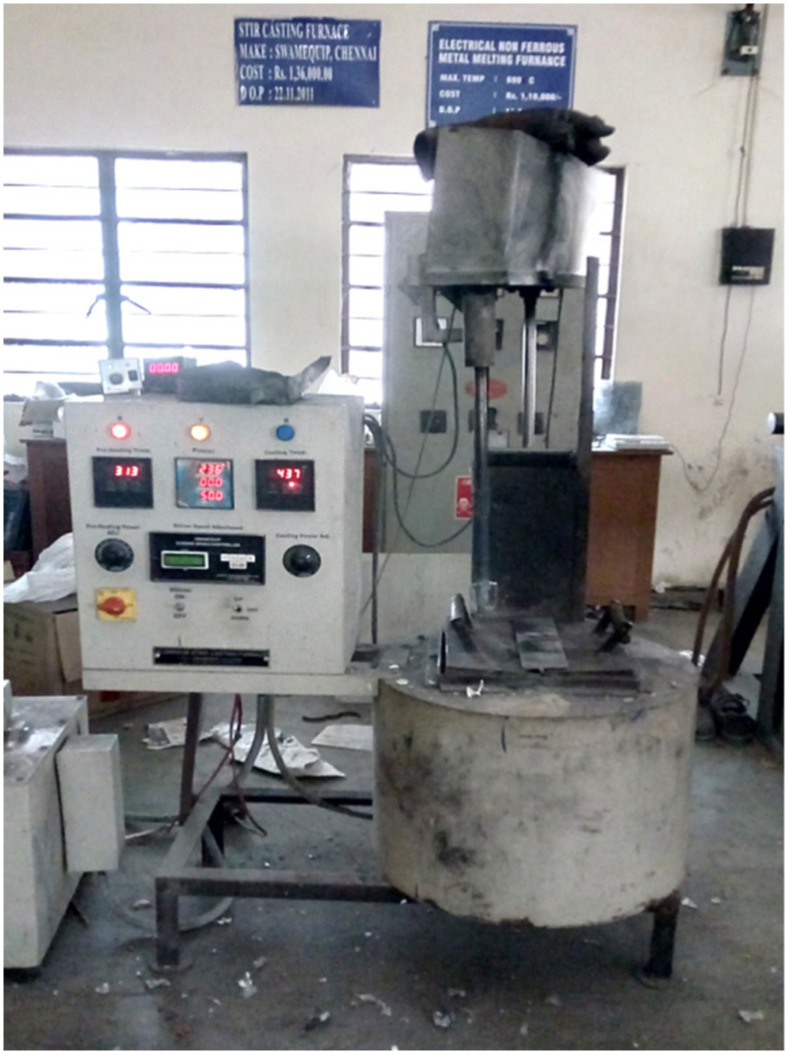
Stir casting setup.

Fabrication of AMMCs was carried out by utilizing a stir casting setup, shown in Figure 1. During this process, the ingots of the base aluminium alloy (Al7068) were heated up to 700 °C in an electronic furnace. Meantime, both ceramic reinforcements (Si_3_N_4_ & BN) were preheated in a muffle furnace at 450 °C to remove the moisture content and produce better bonding with the alloy matrix. After melting the aluminium alloy, the preheated reinforcing particles were added to the alloy melt at a uniform feed rate. With the help of a mechanical stirrer, the mixture was stirred thoroughly at 500 RPM for 10 minutes to attain uniform dispersion of reinforcing particles. Minimum weight fraction (1 wt. %) of magnesium was added to the mixture during the stirring process to improve the wettability [7]. Finally, a thoroughly stirred liquid mixture was poured into a die and allowed to solidify. The details of the nine different AMMC samples, fabricated by varying the weight percentage of both reinforcing materials, are provided in Table 2. Specimens were prepared for conducting metallographic, hardness and dry sliding wear tests from the casted composite materials.

### 2.3. Microstructural Analysis and Hardness Test

A scanning electron microscope (SEM) (Model: JEOL-JSM-IT 200, Manufactured by Jeol Ltd, Tokyo, Japan) was employed to study the microstructure of the developed AMMCs. To carry out microstructural analysis (as per ASTM D3039 standards), specimens of the developed AMMCs were polished using emery sheets and etched using Keller’s etchant. The hardness test was performed on the developed composite specimens as per the ASTM E10 standard; this test method covers the determination of the Brinell hardness of metallic materials by the Brinell indentation hardness principle. Also, this standard provides the requirements like load and size of the indenter for a Brinell testing machine (Model: HR-530, Manufactured by Mitutoyo Corporation, Kanagawa, Japan), and the procedures for performing Brinell hardness tests. [25]. A Brinell hardness tester with 500 kg load and 10 mm hardened steel ball indenter was utilised to conduct the hardness test.

### 2.4. Dry Sliding Wear Study on AMMCs

**Figure 2:**
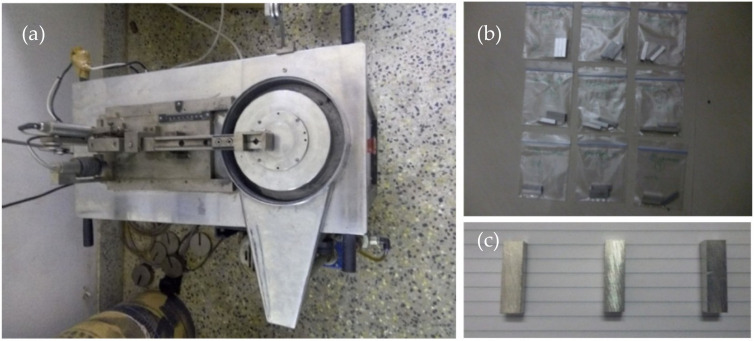
(**a**) Pin-on-disc wear tester setup, (**b**) specimens prepared for wear test, and (**c**) specimens after wear test.

A dry sliding wear experiment was carried out with the help of a pin-on-disc wear tester (Model: DUCOM TR-20LE-PHM 400-CHM 500, Manufactured by Ducom Instruments (India) Pvt. Ltd., Bangalore, India) as per the guidelines provided in the ASTM G99-95 standard. The composite pin specimen was kept against a rotating EN32 steel disc (62 HRC) for a predetermined time during the wear experiment. The composite pin specimens used for the wear experiment were 6 mm × 6 mm × 30 mm. The pin-on-disc wear tester setup and wear specimens are shown in Figure 2. The diameter of the wear track for all the experimental runs was fixed as 100 mm. To get the responses (wear rate and COF) preciously, each wear experimental run was repeated thrice, and the average value was recorded. The weight-loss method was used to calculate the wear rate of the specimens. On completion of each experimental run, the composite specimen and the steel disc were cleaned with acetone; the weight loss of the wear specimens was determined by weighing the specimens before and after the wear experiment with the help of an electronic weighing balance with a resolution of 0.0001 g. The wear rate of the composite pin specimen was obtained with the use of the formula given below [6].

(1) $$\mathrm{wear}\mathrm{rate}=\frac{weight\;loss}{distance}\;g/m$$

The frictional force during each experimental run was obtained through a data acquisition system, and the COF was determined with the help of the following expression [7].

(2) $$\mathrm{COF}=\frac{Frictional\;force}{applied\;load}$$

### 2.5. Plan of Wear Experiments

In this wear experimental work, the wear and friction behaviour of the composite material is governed by five process parameters and their three levels. Hence, a more significant number of experimental runs need to be conducted to investigate the effect of process parameters on the response characteristics. Conducting a larger number of experimental runs is quite tedious and expensive. To overcome this issue, the Design of Experiments (DOE) can be adopted. The effect of the process parameters on the responses can be studied by conducting a minimal number of experimental runs. The Taguchi method is frequently utilised in many engineering applications to reduce the number of experimental runs.

Moreover, it is a promising technique for tribological aspects which have been applied by researchers recently [26,27]. In this context, Taguchi-based DOE methodology was employed in this wear study. The independent control parameters and their levels considered for this experimental work are provided in Table 3. Based on the Taguchi method of DOE, L_27_ orthogonal array design was selected for five parameters with three levels, and the wear experimental runs were conducted accordingly. Minitab 17 software (Software version: 17, 2021, Manufacturer: Minitab Ltd., State College, PA, USA) was employed to design the L_27_ combination. The signal to noise ratio (S/N ratio) was calculated for the response characteristics using the experimental results. The S/N ratio, in general, is used to measure the variation in the response variable relative to the desirable or target value under various noise conditions. The optimum level of each process parameter of the experiment can be identified by selecting a suitable one from the three approaches of S/N ratios, namely, larger the better, nominal the best and smaller the better. The main objective of this experimental work is to reduce wear rate and COF. Hence, the smaller, the better approach was selected for calculating S/N ratios, and the corresponding equation is given below [25].

(3) SN=−10log101n∑i=1nyi2

where n is the total number of experimental runs and $y_{i}$ represents the output of *i*th experimental run.

## 3. Results and Discussion

### 3.1. Microstructural Evolution of AMMCs

**Figure 3:**
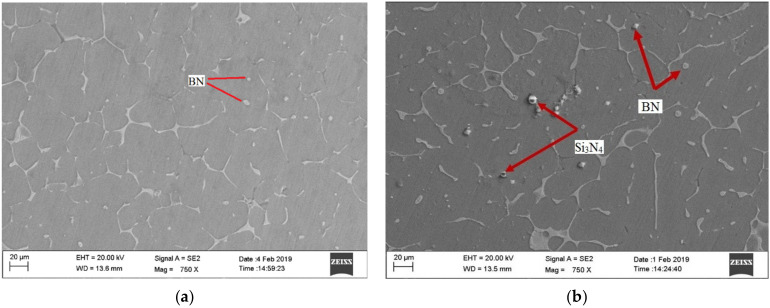
SEM micrograph of AMMC samples: (**a**) S3; (**b**) S8.

In general, the distribution of reinforcing particles in the matrix material highly influences the properties of the fabricated AMMCs. In order to ensure effective reinforcement, the reinforcing particles must be distributed homogeneously in the matrix material. Hence, the distribution of Si_3_N_4_ and BN particles was analysed through SEM. The SEM micrographs shown in Figure 3 show that the reinforcing materials are evenly distributed within the matrix material, and a refined grain structure was gained.

### 3.2. Hardness of AMMCs

The results of the hardness test carried out on the developed composite specimens are provided in Table 4. The hardness values obtained for the composite specimens reveal that both ceramic reinforcing particles (Si_3_N_4_ & BN) significantly improve the hardness of the composite materials. This noteworthy improvement in the hardness of the composites may be due to the presence of comparatively harder ceramic particles in the matrix material [22]. Moreover, the uniform dispersion of ceramic reinforcing particles effectively arrests the movement of grains in the alloy matrix material, thereby reducing the deformation of grains in the alloy matrix, which improves the mechanical behaviour of the composites [28]. The hardness test results also reveal that the increase in the Si_3_N_4_ content in the composites significantly improves their resistance against indentation. Among the fabricated composite samples, Al7068/10 Si_3_N_4_/5BN has a maximum hardness value (108 BHN).

### 3.3. Wear Rate and COF

Table 5 shows the wear rate and COF values for the L_27_ combination. This table also displays the S/N ratios of the response parameters. Based on the S/N ratio values, main effect plots were constructed, and the effect of each input parameter over the responses was identified. 

### 3.4. Effect of Independent Process Parameters on the Wear Rate

**Figure 4:**
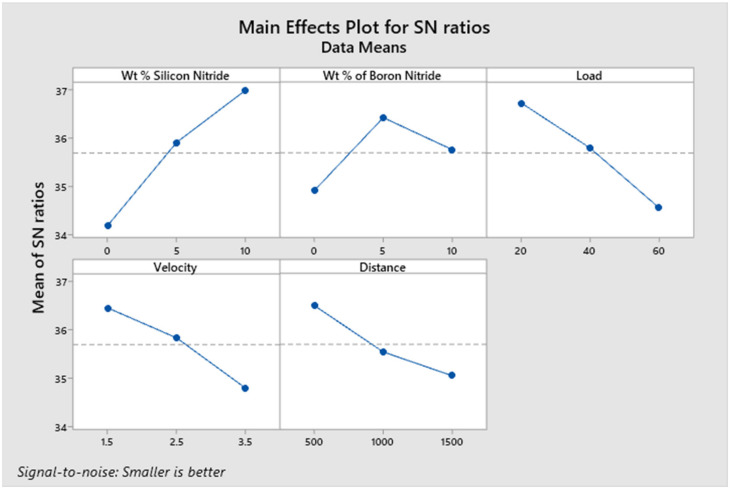
Effect of independent process parameters on the wear rate.

Figure 4 shows the effect of each process parameter on wear rate under dry sliding conditions. The wear rate of the developed composite materials exhibits a decreasing trend with the increase in weight % of Si_3_N_4_. The decreasing trend is owing to the rise in the hardness of the composite materials with the addition of hard Si_3_N_4_ particles [22]. In general, the wear resistance offered by the harder material is much higher than that of the softer material [29]. The hard primary reinforcing material acts as a hurdle for the deformation and, therefore, more energy is required to deform the base alloy matrix material. The addition of BN particles up to 5% improves the wear resistance of the developed composite materials. This improved wear resistance of the composite materials may be attributed to the formation of a smooth BN-rich lubricating layer. This lubricating layer formed between the rubbing surfaces reduces the wear rate [30].

However, on increasing BN content beyond 5 wt. %, the wear rate increases. There is a decrease in the fracture toughness of the composite, as the BN content is increased beyond a certain weight fraction [31,32]; this may be the possible reason for the increase in wear rate, as the content of BN is increased from 5 wt. % to 10 wt. %. It is evident from the main effect plot that the wear rate of the composite pin specimens increases as the applied normal load is increased. The increase in applied load considerably improves the contact pressure between the rubbing surfaces, resulting in increased friction between them [33].

The increased frictional force between the pin specimen and the counter steel disc drastically deforms more material from the pin surface. Therefore, the wear rate increases with an applied normal load. This behaviour of the composite pin is in accordance with Archard wear equation, which indicates a linear relationship between the applied normal load and the wear rate [34]. It is observed from the main effect plot for wear rate that the increase in sliding velocity leads to an increase in wear rate. The heat generation between the rubbing surfaces is more at higher sliding velocities, which may lead to the softening of the composite pin surface. This thermal softening of the pin surface results in increased wear mass loss at higher velocities [35]. The wear experiments also reveal that the wear rate of the developed composite materials increases with the increase in sliding distance. In general, the contact time between the rubbing surfaces increases with the increase in sliding distance. The larger contact time may cause the formation of more wear debris between the rubbing surfaces [36]. These wear debris may exhibit abrasive action on the composite pin surface, and, as a result, the wear rate increases with the increase in running distance. From Figure 4, it is found that the optimum combination of independent input parameters for minimum wear rate is 10 wt. % of Si_3_N_4_, 5 wt. % of BN, 20 N load, 1.5 m/s sliding velocity and 500 m sliding distance.

### 3.5. Effect of Independent Process Parameters on COF

**Figure 5:**
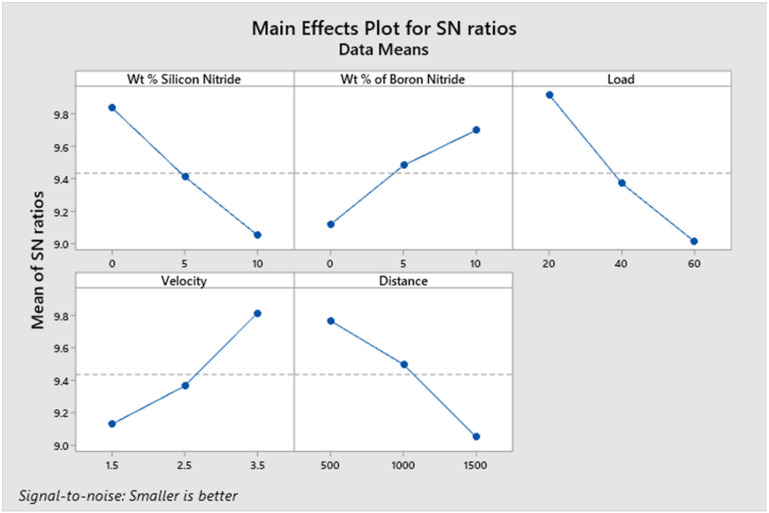
Effect of independent process parameters on COF.

Figure 5 indicates that the COF between the composite pin specimen and the counter disc increases linearly on increasing Si_3_N_4_ content; this may be because as the content of hard ceramic Si_3_N_4_ particles increases, the interlocking force between the sliding surfaces increases, thereby leading to improved resistance to sliding [37]. On the other hand, the increased content of BN decreases COF, which can be attributed to the self-lubricating BN film formed between the surfaces of counter bodies [33]. The protective layer of BN effectively prevents direct contact between the composite pin and the steel disc, and, as a consequence, COF decreases. It is observed that CoF shows an increasing trend on increasing the load from 20N to 60N. This increasing trend of COF is due to the known scenario that the increase in applied load increases the asperity–asperity contacts between the sliding surfaces [38].

Moreover, the thin lubricating film breaking due to increased pressure at the contact interface also leads to increased COF [23]. It is also revealed that the increase in sliding velocity lowers the COF between the surfaces of the counter bodies. The higher sliding velocity leads to thermal softening of the test pin surface due to an increased interface temperature [39]. This thermal softening causes a decrease in the hardness of the pin specimen, resulting in decreased COF. Moreover, the local shear strength of the test specimen is lowered at higher velocities due to increased temperature at the interface, thereby reducing the COF [40]. It is also seen from the main effect plot for COF that the COF increases as the sliding distance is increased. This increase in COF may be attributed to the increased frictional forces between the rubbing surfaces. At longer running distances, the formation of more wear debris between rubbing surfaces leads to increased friction forces, resulting in increased COF [41]. From Figure 5, it is found that the optimum combination of independent input parameters for minimum COF is 0 wt. % of Si_3_N_4_, 10 wt. % of BN, 20 N load, 3.5 m/s sliding velocity and 500m sliding distance.

### 3.6. ANOVA

For this research, ANOVA was used, which is often used for scientific research [42]. ANOVA deals with the amount of the effectiveness of process parameters on response characteristics [43]. The main intention of this method is to determine the process parameters that significantly influence the response characteristics. This analysis was performed at a 5% significance level. The difference between the mean value and the result of each experimental run was used to obtain the sum of squares, which was used to calculate the percent contribution. In general, the calculated *p*-value is utilised to check whether the selected process parameter is statistically significant or not in the 95% confidence interval. ANOVA test results show that the *p*-value of all the parameters considered for this experiment is less than 0.05. This clearly depicts that each parameter has a significant effect on the response characteristics [44,45]. From Table 6, it is also found that wt. % of Si_3_N_4_ particles has a higher contribution percentage (36.60%) on wear rate, followed by applied load (23.01%), sliding velocity (17.52%), wt. % of BN particles (9.71%) and sliding distance (8.22%). Therefore, according to the ANOVA test, wt. % of Si_3_N_4_ particles is the most commanding parameter on wear rate considering the percent contribution. This may be attributed to the increase in the hardness of the developed composites with the addition of hard ceramic particles.

From Table 7, it is observed that load has a higher contribution percentage (29.73%), followed by wt. % of Si_3_N_4_ particles (22.12%), sliding distance (17.84%), sliding velocity (16.93%) and wt. % of BN particles (11.97%). Based on the percent contribution, it is clear that load is the most influencing parameter on COF. This may be attributed to the increased asperity–asperity contacts between the mating surfaces and the breakdown of BN lubricating film with the increase in load. 

### 3.7. Mathematical Modelling

The developed mathematical equations of the wear experiment can be employed to predict response characteristics. The regression equations for the response parameters (wear rate and COF) were developed with the aid of Minitab 17 software based on the values obtained from the wear experimentation. The linear regression equations for the responses are as follows.

Wear rate = 0.00840 − 0.000544 A − 0.000099 B + 0.000109 C + 0.001861 D+ 0.000003 E(4)

COF = 0.30375 + 0.003133 A − 0.002278 B + 0.000906 C − 0.01339 D + 0.000028 E(5)

where, A—wt. % of Si_3_N_4_, B—wt. % of BN, C—applied normal load, D—sliding velocity and E—sliding distance.

### 3.8. Confirmation Experimental Runs

Confirmation experimental runs were performed using a specific set of independent process parameters and levels as given in Table 8, to verify the prediction ability of the developed linear regression equations. The results obtained from the confirmation experimental runs were compared with the values computed using Equations (4) and (5). The comparison between the calculated values and the confirmation experimental values is shown in Table 9. This comparison shows that there is a close match between the experimental values and the predicted values. 

### 3.9. Analysis of Worn-Out Surface of Wear Specimens

**Figure 6:**
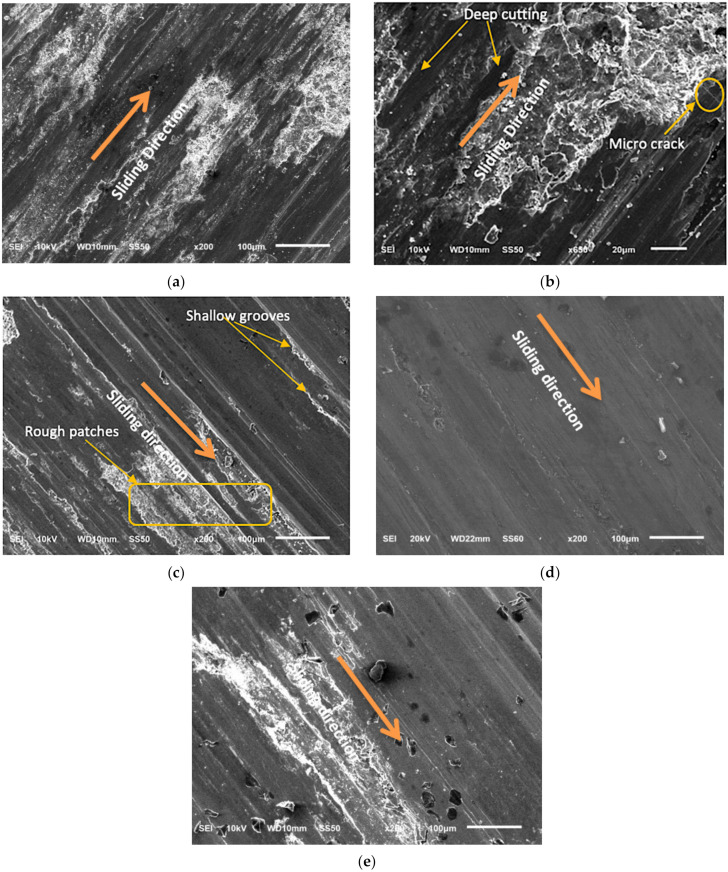
SEM micrograph of AMMC samples. Worn surface of: (**a**) Al7068/10BN at lower magnification; (**b**) Al7068/10BN at higher magnification; (**c**) Al7068/10Si_3_N_4_; (**d**) Al7068/10Si_3_N_4_/5BN; (**e**) Al7068/10Si_3_N_4_/10BN.

Figure 6 shows SEM micrographs of worn surfaces. In general, worn surfaces of the wear test specimens were analysed using SEM to understand the wear behaviour of the composite materials. Figure 6a,b show the worn surface of the Al7068/10BN composite specimen at low and high magnification, respectively, tested under severe operating conditions (higher normal load, sliding velocity and sliding distance). The worn surface exhibits long deformation bands and larger patches along the sliding direction, which indicates the existence of delamination wear. The higher weight fraction of solid lubricant (BN) particles reduces the composite’s resistance to deformation, which in turn causes increased wear loss [23,33]. Deep cutting and micro cracking are visible in Figure 6b. Figure 6c depicts the worn surface of the Al7068/10Si_3_N_4_ composite specimen under severe operating conditions. This SEM micrograph shows shallow grooves and rough patches along the sliding direction. The wear loss is lesser than that of the previous case due to the increased wear resistance of the composite specimen. This improvement in wear resistance may be attributed to the increase in hardness of the composite material with the addition of higher content of hard ceramic reinforcement [22]. The worn-out surface of a composite material reinforced with 10 wt. % of Si_3_N_4_ and 5 wt. % of BN is shown in Figure 6d. This micrograph depicts that the surface deformation is minimal. The presence of a higher amount of Si_3_N_4_ particles greatly improves the wear resistance of the composite material. In addition to that, the solid lubricant-rich film formed between the rubbing surfaces effectively prevents direct contact between the rubbing surfaces [46]. Therefore, the worn-out surface of Al7068/10Si_3_N_4_/5BN has minimal surface damage. The worn surface of the Al7068/10Si_3_N_4_/10BN composite specimen is shown in Figure 6e. This specimen has much-improved wear resistance due to higher Si_3_N_4_ content. However, higher BN content leads to particle detachment, thereby causing the projection of hard phases from the composite pin surface, which is evident from Figure 6e.

## 4. Conclusions

AMMCs were successfully developed in this present work by reinforcing Al7068 with Si_3_N_4_ and BN particles via the stir casting route. Based on the results obtained from the hardness and dry sliding wear tests conducted on the fabricated composite materials, the following conclusions can be drawn:

1. The highest hardness value of the hybrid composite was obtained with the addition of 10 wt. % of Si_3_N_4_ and 5 wt. % of BN.
2. The wear rate of the fabricated composite materials decreases with the addition of Si_3_N_4_ content. However, COF exhibited an increasing trend.
3. The increase in BN content leads to a decrease in COF. However, wear rate initially decreases, up to 5 wt. %, and after that, increases.
4. The minimum wear rate was found with the composite material reinforced with 10 wt. % of Si_3_N_4_ and 5 wt. % of BN.
5. COF increases with the increase in applied load and sliding distance. However, an increase in sliding velocity lowers COF.
6. ANOVA results conclude that wt. % of Si_3_N_4_ (36.60%) has more of a contribution percentage on wear rate followed by load (23.01%).
7. In the case of COF, load (29.73%) is the most influencing parameter, followed by wt. % of Si_3_N_4_ (22.12%).

The results obtained from this study suggest that the developed hybrid composite could be a potential candidate material for various tribological applications, especially in the aerospace and automotive sectors. Hybrid composite materials are used in the aerospace and automotive sectors for their versatile properties like light weight, strength to weight ratio, low cost, ease of structure development and high strength. 

## Figures and Tables

**Table 1:** Chemical composition of Al7068.

Elements	Zn	Mg	Cu	Fe	Zr	Si	Mn	Ti	Cr	Others	Al
Weight %	8.3	3.0	2.4	0.15	0.15	0.12	0.1	0.01	0.05	0.20	balance

**Table 2:** Details of various AMMCs prepared.

Sample Number	S1	S2	S3	S4	S5	S6	S7	S8	S9
Weight percentage of Si_3_N_4_	0	0	0	5	5	5	10	10	10
Weight percentage of BN	0	5	10	0	5	10	0	5	10

**Table 3:** Independent Process Parameters and their levels.

Code	Parameter	Unit	Level		
			1	2	3
A	Weight fraction of Si_3_N_4_	%	0	5	10
B	Weight fraction of BN	%	0	5	10
C	Load	N	20	40	60
D	Sliding velocity	m/s	1.5	2.5	3.5
E	Sliding distance	M	500	1000	1500

**Table 4:** Hardness of the fabricated AMMCs.

Sample No	S1	S2	S3	S4	S5	S6	S7	S8	S9
Hardness (BHN)	97.1	98.6	99.6	104.6	105	107	103	108	107

**Table 5:** L_27_ Taguchi design with response parameters and S/N ratios.

Sl.No	Weight Fraction of Si_3_N_4_ (%)	Weight Fraction of BN (%)	Load (N)	Sliding Velocity (m/s)	Sliding Distance (m)	Wear Rate (mg/m)	S/N Ratio for Wear Rate	COF	S/N Ratio for COF
1	0	0	20	1.5	500	0.0158	36.0269	0.315	10.0338
2	0	0	20	1.5	1000	0.0178	34.9916	0.324	9.7891
3	0	0	20	1.5	1500	0.0187	34.5632	0.344	9.2688
4	0	5	40	2.5	500	0.0164	35.7031	0.309	10.2008
5	0	5	40	2.5	1000	0.0182	34.7986	0.326	9.7356
6	0	5	40	2.5	1500	0.0179	34.9429	0.342	9.3195
7	0	10	60	3.5	500	0.0248	32.1110	0.294	10.6331
8	0	10	60	3.5	1000	0.0232	32.6902	0.314	10.0614
9	0	10	60	3.5	1500	0.0256	31.8352	0.335	9.4991
10	5	0	40	3.5	500	0.0175	35.1392	0.327	9.7090
11	5	0	40	3.5	1000	0.0178	34.9916	0.334	9.5251
12	5	0	40	3.5	1500	0.0228	32.8413	0.354	9.0199
13	5	5	60	1.5	500	0.0148	36.5948	0.356	8.9710
14	5	5	60	1.5	1000	0.0156	36.1375	0.360	8.8739
15	5	5	60	1.5	1500	0.0158	36.0269	0.382	8.3587
16	5	10	20	2.5	500	0.0125	38.0618	0.297	10.5449
17	5	10	20	2.5	1000	0.0147	36.6537	0.313	10.0891
18	5	10	20	2.5	1500	0.0146	36.7129	0.330	9.6297
19	10	0	60	2.5	500	0.0153	36.3062	0.378	8.4502
20	10	0	60	2.5	1000	0.0185	34.6566	0.381	8.3815
21	10	0	60	2.5	1500	0.0185	34.6566	0.402	7.9155
22	10	5	20	3.5	500	0.0109	39.2515	0.309	10.2008
23	10	5	20	3.5	1000	0.0135	37.3933	0.316	10.0063
24	10	5	20	3.5	1500	0.0143	36.8933	0.328	9.6825
25	10	10	40	1.5	500	0.0109	39.2515	0.349	9.1435
26	10	10	40	1.5	1000	0.0133	37.5230	0.355	8.9954
27	10	10	40	1.5	1500	0.0142	36.9542	0.367	8.7067

**Table 6:** Statistical analysis of variance for wear rate.

Source	Degree of Freedom	Sum of Squares	Mean Square	F-Value	*p*-Value	Percent Contribution (%)
wt. % of Si_3_N_4_	2	0.000138	0.000069	59.21	0.000	36.60
wt. % of BN	2	0.000037	0.000018	15.72	0.000	9.71
Load	2	0.000087	0.000043	37.22	0.000	23.01
Sliding velocity	2	0.000066	0.000033	28.35	0.000	17.52
Sliding distance	2	0.000031	0.000015	13.29	0.000	8.22
Error	16	0.000019	0.000001			4.94
Total	26	0.000377				100

**Table 7:** Statistical analysis of variance for COF.

Source	Degree of Freedom	Sum of Squares	Mean Square	F-Value	*p*-Value	Percent Contribution (%)
wt. % of Si_3_N_4_	2	0.004424	0.002212	125.60	0.000	22.12
wt. % of BN	2	0.002395	0.001197	67.99	0.000	11.97
Load	2	0.005947	0.002973	168.84	0.000	29.73
Sliding velocity	2	0.003387	0.001693	96.16	0.000	16.93
Sliding distance	2	0.003568	0.001784	101.31	0.000	17.84
Error	16	0.000282	0.000018			1.41
Total	26	0.020003				100

**Table 8:** Input parameters of confirmation experiment.

Ex. No.	Wt. % of Si_3_N_4_	Wt. % of BN	Load (N)	Sliding Velocity (m/s)	Sliding Distance (m)
1	5	10	30	2.0	600
2	10	5	50	3.0	1200

**Table 9:** Comparison of confirmation test values and predicted values.

Ex. No.	Confirmation Test Wear Rate (mg/m)	Computed Wear Rate (mg/m)	Error (%)	Confirmation Test COF	Computed COF	Error (%)
1	0.0131	0.01348	2.82	0.321	0.31384	2.28
2	0.0164	0.01710	4.09	0.374	0.36242	3.20

## Data Availability

Data is contained within the article.
